# Comparing pregnancy outcomes in a population with natural versus surgical reduction of twin pregnancies: a retrospective cohort study

**DOI:** 10.3389/fendo.2025.1639188

**Published:** 2026-04-02

**Authors:** Yuanxue Jing, Jiawei Gao, Xuzhao Gu, Yi Wan, Xiaoxia He, Xiaoling Ma, Haofei Shen

**Affiliations:** 1 The First Hospital of Lanzhou University, Lanzhou, China; 2 The First Clinical Medical College of Lanzhou University, Lanzhou, China

**Keywords:** spontaneous reduction, twin disappearance syndrome, pregnancy outcome, retrospective cohort study, *in vitro* fertilization

## Abstract

**Objective:**

To compare the cycle of initial twin pregnancy with vanishing twin syndrome (VTS), the cycle of initial twin pregnancy after surgical reduction, and the live birth outcome of initial singleton pregnancy. To provide clinical evidence for confirming that vanishing twin syndrome (VTS) and surgical reduction may affect obstetric and perinatal outcomes.

**Methods:**

A retrospective study was conducted on patients diagnosed with MFPR and VTS at the Reproductive Medicine Hospital of the First Hospital of Lanzhou University from January 2017 to December 2021. A total of 1,796 singleton patients were ultimately included, comprising 271 patients with naturally reduced twin pregnancies, 84 patients with surgically reduced twin pregnancies, and 1,441 patients with singleton pregnancies after IVF/ICSI-assisted conception. A comparison was made on the clinical characteristics and pregnancy outcomes of the three groups.

**Results:**

The rates of preterm birth and low birth weight in the MFPR group were significantly higher than those in the VTS group and the control group (P < 0.05). The miscarriage rate in the MFPR group was significantly higher than that in the VTS group (P < 0.05), but there was no statistically significant difference compared with the control group (P > 0.05). In contrast, the VTS group showed comparable gestational age and birth weight to the control group, with an even lower miscarriage rate. Within the MFPR cohort, Monochorionic diamniotic twin pregnancy (MDT) pregnancies had a higher risk of miscarriage than Dichorionic diamniotic twin pregnancy (DDT) pregnancies (P<0.05). ROC analysis indicated that serum β-hCG levels on day 14 post-transfer had value in distinguishing VTS from initial singletons (AUC = 0.75), with a cutoff of 837 mIU/ml.

**Conclusions:**

The VTS population may have better pregnancy outcomes than the MFPR population and the β-hCG level has certain clinical application value. At the same time, the MFPR in DDT pregnancy is more challenging than that in MDT pregnancy.

## 1 Introduction

Infertility is a global problem with a prevalence rate of 15% to 20.0% (1). *In vitro* fertilization and embryo transfer (IVF-ET) is the main treatment option, but it also leads to problems such as multiple pregnancies (2). Multiple pregnancies after IVF-ET may jeopardize maternal and fetal safety and affect clinical outcomes. Spontaneous pregnancy reduction (SPR) refers to the natural reduction of embryos during multiple pregnancies, where the number of fetuses delivered is less than the number of embryos at the beginning of pregnancy, and most occur within 12 weeks of gestation (3). Luo et al. showed that the incidence of spontaneous abortion after IVF-ET was 17.1% (4). In contrast, the prevalence of spontaneous attenuation was as high as 50% in 38 trimester pregnancies in a study by Manzur et al (5). By comparison, the incidence of spontaneous abortion in women with assisted reproduction twin pregnancies is more accurate because of the need for continuous ultrasound monitoring after assisted reproduction techniques.

Levi (6) first referred to the early loss of one gestational sac in a twin pregnancy as vanishing twin syndrome (VTS) in 1976.In assisted reproduction-assisted pregnancies, the incidence can range from 12% to 30% and may even be as high as 38% (7). The high prevalence of VTS and its impact on pregnancy outcomes has become a topic of concern in the reproductive field. The exact etiology of spontaneous abortions is currently unknown. Several etiologies have been proposed, including placental degeneration, chromosomal abnormalities in embryo loss, inappropriate implantation site, placental “crowding”, intrauterine hemorrhage, and chronic maternal disease. The increase in the number of embryos transferred in assisted reproduction is thought to be an important factor in natural attrition (8). Studies (9) have shown that the incidence of spontaneous reduction is positively correlated with the number of gestational sacs at the onset of pregnancy. Dickey et al (10) reported a spontaneous reduction rate of more than 50% in 155 triplet and higher order pregnancies. Sukur et al (11) reported that the risk of spontaneous reduction of multiple pregnancies with *in vitro* fertilization doubled with each additional embryo transferred.

In addition to this, spontaneous abortions are also related to the age of the pregnant woman and the method of assisted conception (9). Ross (6) and others have shown that the incidence of spontaneous abortions is significantly higher in women older than 30 years of age than in those younger than 30 years of age. A study by La Sala et al (12) revealed that the incidence of spontaneous attrition in twin pregnancies after IVF-ET assisted conception was more pronounced in the group of women of advanced age. Infertility-related treatments may also affect the risk of VTS. Dickey et al (10) reported that spontaneous abortions after induced ovulation occurred less frequently than spontaneous ovulation. The further demonstration by Marton et al (13) that the IVF-ICSI procedure reduces the risk of VTS compared to natural pregnancies suggests that the artificial selection procedure for morphologically normal embryos reduces the rate of chromosomal defects in the fetus, and thus reduces the rate of VTS after IVF-ICSI. Harris et al (14) found that tubal infertility was also a risk factor for VTS, whereas male factor infertility appeared to be a protective factor.

The impact of VTS on obstetric and perinatal outcomes is a controversial subject. Dickey et al (10) reported that, compared with unreduced pregnancies with the same number of fetuses at birth, multiple pregnancies with spontaneous reduction early in gestation had earlier delivery and lower birth weights. On the other hand, Romanski et al. (15) showed that VTS pregnancies conceived through IVF had similar perinatal compared to singleton pregnancies outcomes, including preterm labor rates and birth weight. Several studies have shown increased mortality in VTS compared to singleton pregnancies (16, 17). Romanski et al (15) also demonstrated that the that VTS pregnancies are comparable to IVF singleton pregnancies in terms of obstetric outcomes, including hypertensive disorders of pregnancy, postpartum hemorrhage, and incidence of initial cesarean delivery. On the other hand, an earlier retrospective cohort by Chasen et al (16) study found a higher incidence of preeclampsia in VTS pregnancies compared to singleton pregnancies.

When a twin pregnancy is diagnosed following ART, clinicians and patients face a dilemma: should they opt for an immediate MFPR to mitigate the risks of twin pregnancy, or should they adopt a ‘wait-and-see’ approach in anticipation of a potentially more benign spontaneous reduction (VTS)? Current evidence directly comparing these two pathways to a singleton birth is limited and conflicting. Our study directly addresses this clinical equipoise. By demonstrating that pregnancy outcomes after VTS are significantly better than those after MFPR and are comparable to initial singletons, our findings provide robust evidence to support a more conservative management strategy in selected cases. This can potentially help avoid unnecessary invasive procedures and their associated risks for a significant number of patients.While previous studies have broadly compared reduced and unreduced pregnancies, our study provides novel insights by specifically examining the role of chorionicity in MFPR outcomes. We found that the risk of miscarriage after fetal reduction is significantly higher in monochorionic diamniotic (MDT) pregnancies than in dichorionic diamniotic (DDT) pregnancies, and this difference has direct clinical implications for risk counseling and surgical planning.

## 2 Methods

### 2.1 Data sources and patient selection

A retrospective cohort was established using data from the centralized electronic medical record and IVF database of our reproductive medicine center. To ensure data integrity, a stepwise patient identification and verification process was employed: ① All IVF/ICSI cycles achieving clinical pregnancy between January 2017 and December 2021 were initially screened. ② The VTS group was identified by cross-referencing the laboratory database with early ultrasound records. Inclusion required a clear ultrasound report confirming the presence of two gestational sacs or fetal heartbeats in the first trimester (6-8 weeks), followed by a subsequent report documenting the disappearance of one fetus, culminating in a singleton live birth.③ The MFPR group was identified through the operative record system, selecting all patients who underwent reduction from a twin to a singleton pregnancy and delivered a single neonate. ④ The control group consisted of randomly selected patients from the same period whose first ultrasound confirmed a singleton gestation and who subsequently had a singleton live birth.

Data on clinical characteristics and outcomes for all eligible patients were independently extracted by two researchers using a pre-designed data collection form. Any discrepancies were resolved by consensus or by consulting a third senior investigator.

The study was approved by the First Hospital of Lanzhou University review boards. The inclusion criteria were that (1) Clinical pregnancy after assisted reproductive technology;(2) Complete records of pregnancy outcomes and newborn births; (3) Follow-up until delivery of the fetus, with no missed visits; (4) Natural delivery of a live fetus after a clinical pregnancy. The exclusion criteria include (1) patients with unreduced twin pregnancies and those with unreduced multiple pregnancies; (2) history of recurrent miscarriages, and patients with uterine anomalies; (3) Those who terminate pregnancy on their own due to systemic diseases;(4) The woman has hypertension, diabetes and family history before pregnancy;(5) Cycle of preimplantation genetic diagnosis of embryos.

### 2.2 Statistical analysis

The primary outcome measure include clinical characteristics and pregnancy outcomes. Clinical characteristics were age, body mass index, type of infertility, IVF parameters (number of oocytes retrieved, number of embryos transferred, serum β-hCG, serum estradiol (E2), serum progesterone (P)). The pregnancy outcomes are week of gestation, birth weight, preterm birth rate, miscarriage rate, and low birth weight rate.

Statistical analysis was performed using IBM SPSS 22.0 and ROC curves were performed using GraphPad Prism 6.01. Continuous variables were expressed as mean ± standard deviation (SD) and compared using t-test. If normality was not satisfied, comparisons were made using the Mann-Whitney U test. Data on categorical variables were statistically described using frequency counts (frequencies), and χ2 tests were used to compare differences between groups. P < 0.05 was considered statistically significant.

## 3 Results

### 3.1 Patient characteristics

**Figure 1:**
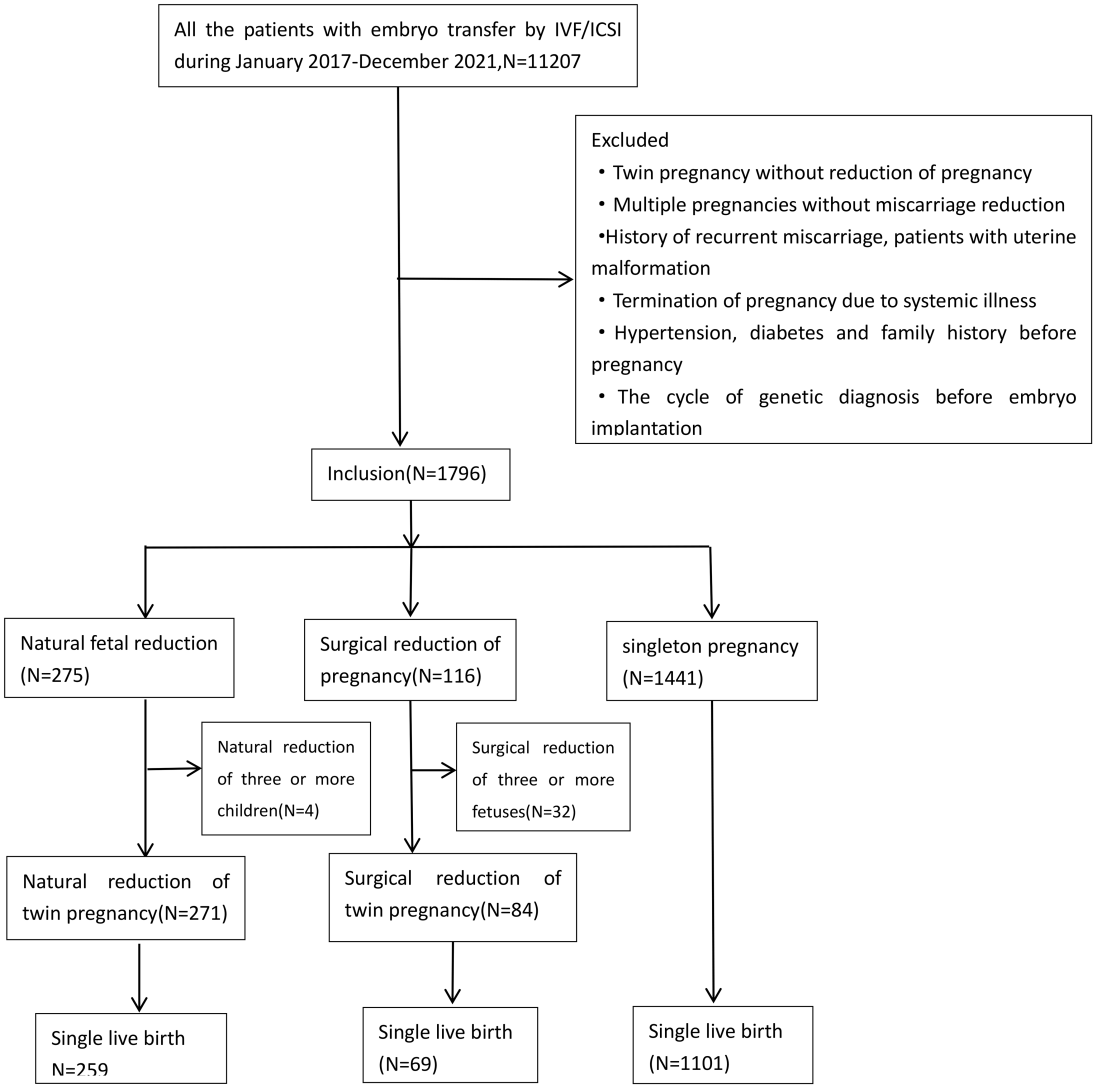
Retrospective analysis of the roadmap for incorporating research subjects.

The final enrollment of 1796 cases included 271 cases of Twin Fetal Disappearance Syndrome and 84 cases of Twin Fetal Gestation Reduction (TFGR), and 1441 IVF/ICSI singleton pregnancies in the control group **Figure 1**.

The baseline clinical characteristics of the VTS group (n=271), the MFPR group (n=84) and the control group (n=1441) are shown in **Table 1**. There was little difference between the two groups in terms of age, type of infertility, body mass index, number of eggs obtained, method of fertilization, and endometrial thickness on the day of transplantation (P>0.05). The fresh embryo transfer rate was significantly lower in the MFPR group than in the VTS (P < 0.05). The number of embryos transferred in the MFPR group was higher than that in the VTS group (P<0.05). On the 14th day after embryo transfer, the serum β-hCG level in the MFPR group was significantly higher than that in the VTS and control groups (P<0.05).

**Table 1:** The characteristics of cycles in different groups.

Characteristics	VTS	MFPR	Control group	VTS vs. MFPR P value	MFPR vs. control group P value	VTS vs. control group P value
Number of patients	271	84	1441			
Age(y)	30.46±4.56	31.13±4.10	31.01±4.45	0.19	0.58	0.13
Infertility type				0.64	0.88	0.26
Primary infertility incidence rate	169(62.36)	50(59.52)	846(58.71)			
Incidence of secondary infertility	102(37.64)	34(40.48)	595(41.29)			
BMI	22.38±3.10	22.05±2.97	22.31±3.05	0.62	0.56	0.99
Number of retrieved oocytes	15.72±8.11	15.70±7.31	14.68±6.91	0.60	0.15	0.18
Number of embryos transferred	2.10±0.32	2.23±0.42	2.17±0.47	0.01*	0.30	0.02*
Fertilization method				0.53	0.15	0.23
IVF(%)	159(58.67)	46(54.76)	901(62.53)			
ICSI(%)	112(41.33)	38(45.24)	540(37.47)			
Transplantation method				0.00*	0.00*	0.13
Fresh cycle transplantation(%)	126(46.49)	18(21.43)	599(41.57)			
Thawing cycle transplantation (%)	145(53.51)	66(78.57)	842(58.43)			
Endometrial thickness (mm)	10.58±2.15	10.70±2.12	10.26±2.09	0.63	0.11	0.06
after 14 days of transplantation						
hcg (mIU/ml)	1855±1550	2239±1601	929.5±1053	0.02*	0.00*	0.00*
E2 (pg/ml)	712.6±736.0	798.0±747.8	604.3±636.3	0.22	0.02*	0.02*
P (ng/ml)	33.62±17.98	34.94±17.64	38.60±60.25	0.51	0.91	0.16

VTS, vanishing twin syndrome with singleton pregnancy.

MFPR, Reduced multiple pregnancies with singleton pregnancies.

Control group, singleton.

### 3.2 Comparison of pregnancy outcomes in VTS, MFPR, and control groups

Birth weight and gestational week of delivery were significantly lower in the MFPR group than in the VTS group and the control group (P<0.05). The rates of miscarriage, preterm labor and low birth weight were significantly higher in the MFPR group than in the VTS group (P<0.05). There was no significant difference in the rate of very low birth weight between the VTS and MFPR groups (P>0.05) **Table 2**.

**Table 2:** Comparison of pregnancy outcomes among VTS, MFPR, control group.

Characteristics	VTS	MFPR	Control group	VTS vs. MFPR P value	MFPR vs. Control group P value	VTS vs. Control group P value
Number of patients	271	84	1441			
The gestational age	38.64±2.14	37.57±2.65	38.90±1.80	0.00*	0.00*	0.15
Birth weight	3154±626.8	2823±658.2	3232±511.9	0.00*	0.00*	0.19
Abortion rate (n,%)	12(4.43)	13(15.48)	340(23.59)	0.00*	0.09	0.00*
Premature birth rate (n,%)	37(13.65)	20(23.81)	105(7.29)	0.03*	0.00*	0.00*
Low birth weight rate (n,%)	26(9.59)	20(23.81)	67(4.65)	0.00*	0.00*	0.00*
Extremely low birth weight rate(n,%)	5(1.85)	2(2.38)	4(0.28)	0.76	0.04*	0.01*

### 3.3 Comparison of pregnancy outcomes between dichorionic diamniotic and monochorionic diamniotic groups

There was no difference in gestational week of delivery, birth weight, preterm birth rate, and low birth weight rate between the two groups (P>0.05). The abortion rate was statistically higher in the MDT group than in the DDT group (p < 0.05) **Table 3**. Curve fitting and threshold effect analysis of HCG on spontaneous abortion after IVF-ET.

**Table 3:** Comparison of pregnancy outcomes between dichorionic diamniotic twin pregnancy undergoing fetal reduction surgery and monochorionic diamniotic twin pregnancy undergoing fetal reduction surgery.

Characteristics	TCT	DCT	P value
Number of patients	50	34	
The gestational age	37.91±2.25	36.94±3.21	0.42
Birth weight	2827±520.8	2816±868.9	0.98
Abortion rate (n,%)	4(8)	9(26.47)	0.03*
Premature birth rate (n,%)	10(20)	10(29.41)	0.32
Low birth weight rate (n,%)	12(24)	8(23.53)	0.96

DDT, Dichorionic diamniotic twin pregnancy undergoing fetal reduction surgery.

MDT, Monochorionic diamniotic twin pregnancy undergoing fetal reduction surgery.

**Figure 2:**
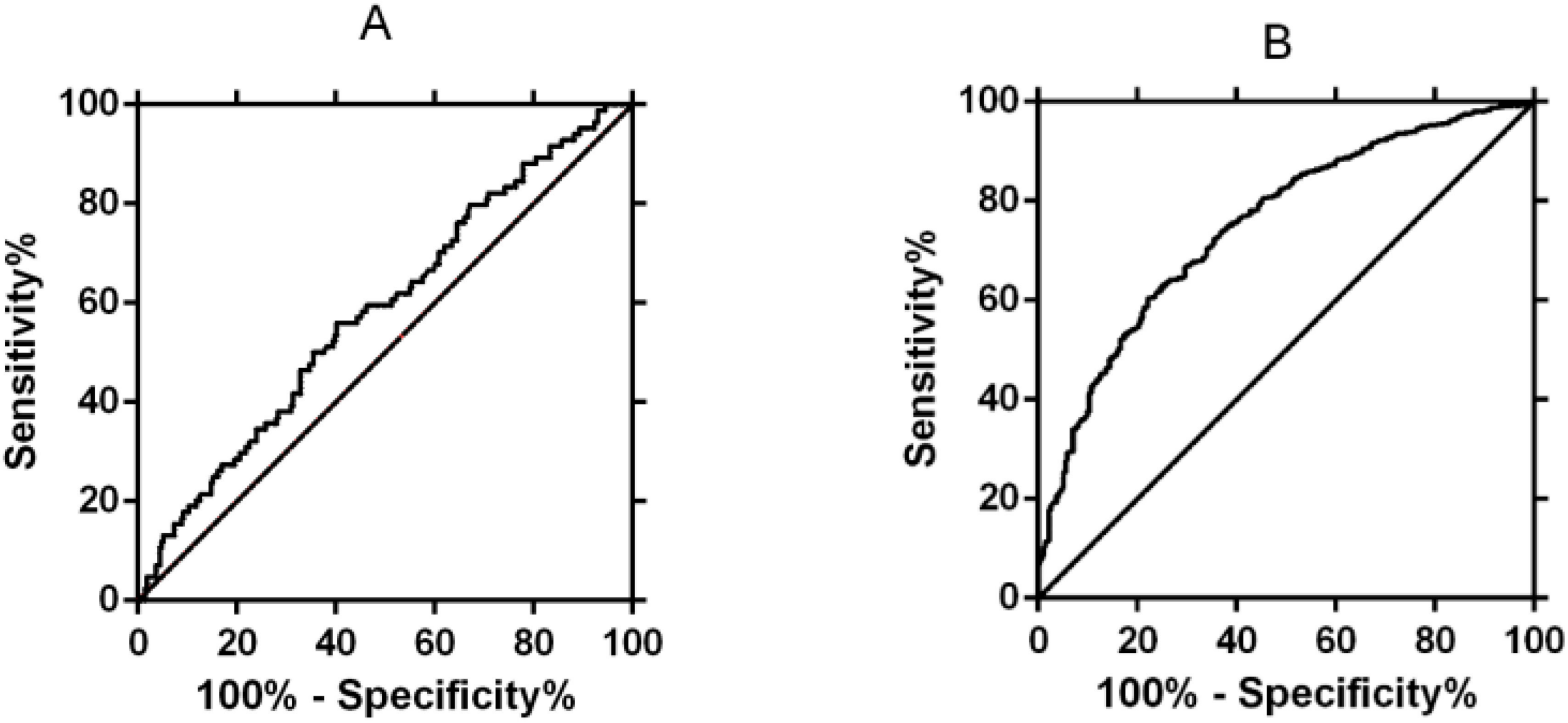
Curve fitting of β-hCG on natural fetal reduction after IVF-ET. **(A)** ROC curve analysis of serum β-hCG levels differentiating VTS from MFPR. **(B)** ROC curve analysis of serum β-hCG levels differentiating VTS from initial singleton pregnancies.

Shows the ROC curve analysis of serum β-hCG-ET14d between the VTS and MFPR groups, with an area under the ROC curve (AUC) of 0.58, 95% confidence interval (0.51-0.65), and a cut-off value of 1687 mIU/ml for β-hCGET14d **Figure 2A**. Shows the ROC curve analysis of serum β-hCG-ET14d between the VTS group and the control group, with an area under the ROC curve (AUC) of 0.75, 95% confidence interval (0.72-0.78), and β-hCG-ET14d cutoff value of 837 mIU/ml **Figure 2B**.

## 4 Discussion

With changes in fertility policies and the widespread use of assisted reproductive technologies, the rate of multiple pregnancies has risen significantly. Multiple pregnancies may lead to serious pregnancy complications and increased incidence of miscarriage and preterm labor. The main mechanism is ischemia of the uterus and placenta due to rapid enlargement of the uterus. The increase in blood volume is more pronounced in women with multiple pregnancies than in singleton pregnancies, which increases the incidence of intrauterine growth restriction and low birth weight (18). Clinicians need to inform patients of the risks of multiple pregnancies and take proactive therapeutic measures to prevent complications, such as elective multifetal pregnancy reduction (MFPR). The use of MFPR in twin pregnancies is more established, with significant improvements in maternal complications, comorbidities, and perinatal outcomes in singleton pregnancies after reduction compared to unreduced twin pregnancies (19). It has also been suggested that there is an increase in the rate of miscarriage after fetal reduction and that some of the miscarriages occur in association with fetal reduction maneuvers (20).

Indications for MFPR include fetal structural abnormalities, chromosomal abnormalities, severe maternal and fetal comorbidities, and social factors. MFPR is an invasive procedure that increases the risk of infection and leads to an increased rate of miscarriage. A retrospective study found that MFPR increased the risk of miscarriage compared to conservative treatment (21). Possible reasons for the increased rate of miscarriage after fetal reduction may include trauma or infection caused by surgical manipulation; the decompensated fetus and its appendages cause an inflammatory response in the mother; leakage of drugs into the amniotic cavity during injection of potassium chloride; incomplete inter-fetal traffic vessel blockage, etc. (22). However, some studies have shown that the effect of MFPR on abortion rates is minimal (23). A study comparing the obstetric outcomes of 1, 000 pregnancies in which triplets were reduced to twins versus singleton pregnancies found that surgical reduction was also associated with higher birth weights and lower rates of preterm labor (24). Differences between studies may be due to different populations, sample sizes, and methods of surgical fetal reduction. The outcome differences between MFPR and VTS: Surgical intervention versus natural selection. In this study, the poorer pregnancy outcomes in the MFPR group might be directly related to the invasiveness of the surgery itself. Surgical procedures may cause uterine contractions, inflammatory responses, and even infections, thereby increasing the risks of miscarriage and premature birth (21, 22). In contrast, VTS is regarded as a natural selection process. Its core mechanism typically involves one of the embryos naturally dying due to inherent chromosomal abnormalities such as aneuploidy, poor implantation location, or poor placental development (8, 12). Therefore, VTS is essentially a “survival of the fittest” process, and the remaining fetus is usually the more vigorous one. This explains why the outcomes of the VTS group are closer to those of the initial singleton pregnancy group and even show better results in terms of the miscarriage rate. Our findings support the view that natural fetal reduction is superior to artificial intervention in avoiding subsequent risks.

Selection of Target Embryos and Associated Risks:In our study, all MFPR surgeries followed the standardized procedures established by our center. In the absence of pre-implantation genetic testing, the selection of the target embryos was mainly based on a comprehensive set of ultrasound morphological criteria. This included choosing the embryo with the smallest crown-rump length (CRL), the embryo with the least amniotic fluid volume, or the embryo with morphological abnormalities (such as abnormal yolk sac or weak fetal heart beat) (20, 22). Additionally, the feasibility and safety of the operation were also important considerations, such as prioritizing the selection of the embryo closest to the puncture path to minimize interference with the retained gestational sac.

However, we must admit that relying solely on ultrasound morphological assessment is not foolproof. There is a theoretical risk that an embryo with normal chromosomes but temporary developmental delay may be mistakenly selected as the target for reduction, while an embryo with chromosomal abnormalities but seemingly normal early development may be retained. This is precisely the fundamental difference between surgical reduction and natural reduction: the latter is determined by natural physiological mechanisms, while the former relies on clinical judgment and involves uncertainty. To reduce this risk, our center conducts multiple and detailed ultrasound evaluations before the surgery, and usually recommends performing the surgery when the gestational age is slightly larger (such as 11-14 weeks), and conducting a preliminary assessment of the fetus through ultrasound screening (such as NT examination). Future research that introduces non-invasive prenatal testing technologies may provide more genetic information for this decision, thereby optimizing the selection process.

It has been shown (25) that the incidence of fetal and neonatal complications varies between chorionicity and the outcome depends on chorionicity and not on amnioticity. Monochorionic diamniotic (MCDA) is a special type of monozygotic twins whose main characteristic is that the two fetuses share a single placenta, Extensive vascular anastomosis exists in the vast majority of placentas, leading to intrauterine fetal death or severe neonatal complications due to hemodynamic imbalance between the two fetuses and unequal placenta partitioning thereby potentially resulting in complications such as twin fetal transfusion syndrome, selective fetal growth restriction, arterial reverse serial perfusion. Multiple pregnancy reduction improves fetal prognosis in the above complications. This study found that after undergoing reduction surgery for monochorionic diamniotic twin pregnancies, the miscarriage rate was higher than that of patients with dichorionic diamniotic sacs. Women with DCT pregnancies delivered on average 2 weeks earlier than those with TCT pregnancies (26), which is consistent with the findings of the present study. There have been few reports of MFPR in patients with DCT or TCT pregnancies compared to VTS, but studies have found improved outcomes in both DCT and TCT pregnancies when patients opt for MFPR (22).

The predictive value of serum β-hCG:Our study also revealed that the serum β-hCG level at 14 days after transplantation differed among the VTS, MFPR, and control groups, and the critical value for predicting VTS was determined. Although the area under the ROC curve (AUC = 0.58) indicated that its ability to distinguish VTS from MFPR was limited, it showed good value in differentiating VTS from the initial singleton pregnancy (AUC = 0.75). This suggests that a lower early β-hCG level may indicate a higher risk of subsequent natural fetal loss. However, it must be emphasized that β-hCG can only serve as an auxiliary early warning indicator, and the diagnosis of VTS still requires subsequent ultrasound examinations.

In this study, we found that 5.5% (271/4974) of singleton births after IVF/ICSI originated from twin vanishing syndrome. Previous studies have reported (10) that VTS occurs in 36% of twin pregnancies and 53% of triple pregnancies. A retrospective study found that VTS occurred in 9% (264 out of 2829) of intracytoplasmic sperm injection pregnancies and was associated with a lower rate of miscarriage in early pregnancy (27). Many studies have reported obstetric outcomes after ART in patients with VTS and singleton, but the results are controversial. One study found that VTS, as a form of natural selection, had similar perinatal outcomes to singleton pregnancies (28). However, other studies have found that VTS is associated with a higher risk of adverse obstetric outcomes, such as gestational diabetes, low Apgar scores, and perinatal mortality (17, 27). In this study, there was no difference in gestational week of delivery and birth weight in the VTS group compared to the control group, but the miscarriage rate was lower in the VTS group than in the singleton control group. This may indicate that VTS, as a natural selection, has a better pregnancy outcome than singleton. VTS may alter the placental environment and lead to adverse pregnancy outcomes. In this study, the rate of preterm labor and low birth weight was found to be higher in the VTS group than in those with singleton pregnancies, which is consistent with Harris (14).

This study has several limitations. Firstly, as a single-center retrospective study, although we made efforts to control for known confounding factors, we were unable to obtain and analyze data such as embryo quality scores and detailed miscarriage histories of the patients, which may introduce potential residual confounding that could affect the interpretation of the results. Secondly, data on some patients’ pregnancy complications were missing due to delivery in other hospitals. Additionally, regarding the specific details of the selection of the reduction target, as mentioned earlier, it mainly relied on morphological criteria, which has inherent limitations. Future multi-center, prospective studies that integrate embryonic genetic information with detailed clinical data will be able to more accurately assess the independent risks of reduction surgery and further optimize the selection strategy for target embryos.

## 5 Conclusion

The results of this study suggest that women with DCT who undergo MFPR have a higher risk of miscarriage compared to TCT. Selective single-embryo transfer may be a better option, and one study (11) found that the cumulative live birth rate in the single-embryo transfer group was similar to that of the double-embryo transfer group. The number of embryos transferred in the MFPR group was higher than that in the VTS and control groups. Therefore, single embryo transfer is still recommended even for patients with repeated transfer failures, premature ovarian failure and advanced infertility.

## Data Availability

The original contributions presented in the study are included in the article/supplementary material. Further inquiries can be directed to the corresponding author.

## References

[1] Mazzilli R Rucci C Vaiarelli A . Male factor infertility and assisted reproductive technologies: indications, minimum access criteria and outcomes. J Endocrinol Invest. (2023) 46:1079–85. doi: 10.1007/s40618-022-02000-4, PMID: 36633791 PMC10185595

[2] Katler QS Kawwass JF Hurst BS . Vanquishing multiple pregnancy in *in vitro* fertilization in the United States—a 25-year endeavor. Am J Obstetr Gynecol. (2022) 227:129–35. doi: 10.1016/j.ajog.2022.02.005, PMID: 35150636

[3] Bosch E Labarta E Crespo J . Circulating progesterone levels and ongoing pregnancy rates in controlled ovarian stimulation cycles for *in vitro* fertilization: analysis of over 4000 cycles. Hum Reprod. (2010) 25:2092–100. doi: 10.1093/humrep/deq125, PMID: 20539042

[4] Luo L Cai B Jie H-y . Influence of spontaneous fetal reduction on dichorionic diamniotic twin pregnancy outcomes after *in vitro* fertilization: a large-sample retrospective study. J Maternal-Fetal Neonatal Med. (2018) 32:1826–31. doi: 10.1080/14767058.2017.1419178, PMID: 29251184

[5] Manzur A Goldsman MP Stone SC . Outcome of triplet pregnancies after assisted reproductive techniques: How frequent are the vanishing embryos? Fertil Steril. (1995) 63:252–7. doi: 10.1016/S0015-0282(16)57350-6, PMID: 7843426

[6] Carroll SGM Soothill PW Abdel-Fattah SA . Prediction of chorionicity in twin pregnancies at 10–14 weeks of gestation. BJOG: Int J Obstetr Gynaecol. (2003) 109:182–6., PMID: 11905430 10.1111/j.1471-0528.2002.01172.x

[7] Abdalla HI Burton G Kirkland A Johnson MR Leonard T . Age, pregnancy and miscarriage: uterine versus ovarian factors. Hum Reprod. (1993) 8:1512–7. doi: 10.1093/oxfordjournals.humrep.a138289, PMID: 8253944

[8] La Sala GB Nucera G Gallinelli A . Spontaneous embryonic loss following *in vitro* fertilization: Incidence and effect on outcomes. Am J Obstetr Gynecol. (2004) 191:741–6. doi: 10.1016/j.ajog.2004.03.076, PMID: 15467533

[9] Maheshwari A Stofberg L Bhattacharya S . Effect of overweight and obesity on assisted reproductive technology—a systematic review. Hum Reprod Update. (2007) 13:433–44. doi: 10.1093/humupd/dmm017, PMID: 17584821

[10] Dickey RP Taylor SN Lu PY . Spontaneous reduction of multiple pregnancy: Incidence and effect on outcome. Am J Obstetr Gynecol. (2002) 186:77–83. doi: 10.1067/mob.2002.118915, PMID: 11810089

[11] Sukur YE Altun T Pal L . Predictors of spontaneous reduction in multiple pregnancies conceived following assisted reproductive technology. Eur J Obstetr Gynecol Reprod Biol. (2012) 162:174–7. doi: 10.1016/j.ejogrb.2012.02.031, PMID: 22498231

[12] Vanneste E Voet T Le Caignec C . Chromosome instability is common in human cleavage-stage embryos. Nat Med. (2009) 15:577–83. doi: 10.1038/nm.1924, PMID: 19396175

[13] Márton V Zádori J Keresztúri A . Associated perinatal determinants of vanishing twin pregnancies achieved by *in vitro* fertilization vs. spontaneous conception. Arch Gynecol Obstetr. (2020) 301:491–8. doi: 10.1007/s00404-020-05448-y, PMID: 32025846

[14] Harris AL Sacha CR Basnet KM . Vanishing twins conceived through fresh *in vitro* fertilization. Obstetr Gynecol. (2020) 135:1426–33. doi: 10.1097/AOG.0000000000003888, PMID: 32459435

[15] D’Antonio F Odibo AO Prefumo F . Weight discordance and perinatal mortality in twin pregnancy: systematic review and meta-analysis. Ultrasound Obstetr Gynecol. (2018) 52:11–23. 10.1002/uog.18966 29155475

[16] Chasen ST Luo G Perni SC . Are *in vitro* fertilization pregnancies with early spontaneous reduction high risk? Am J Obstetr Gynecol. (2006) 195:814–7., PMID: 16949417 10.1016/j.ajog.2006.06.022

[17] Evron E Sheiner E Friger M . Vanishing twin syndrome: is it associated with adverse perinatal outcome? Fertil Steril. (2015) 103:1209–14., PMID: 25772775 10.1016/j.fertnstert.2015.02.009

[18] Sato Y Ishii K Yokouchi T . Incidences of feto-fetal transfusion syndrome and perinatal outcomes in triplet gestations with monochorionic placentation. Fetal Diagnosis Ther. (2016) 40:181–6. doi: 10.1159/000443610, PMID: 26760043

[19] Greenberg G Bardin R Danieli-Gruber S . Pregnancy outcome following fetal reduction from dichorionic twins to singleton gestation. BMC Pregnancy Childbirth. (2020) 20. doi: 10.1186/s12884-020-03076-7, PMID: 32620088 PMC7333296

[20] Kim MS Kang S Kim Y . Transabdominal fetal reduction: a report of 124 cases. J Obstetr Gynaecol. (2020) 41:32–7. doi: 10.1080/01443615.2019.1677577, PMID: 32705924

[21] Papageorghiou AT Avgidou K Bakoulas V . Risks of miscarriage and early preterm birth in trichorionic triplet pregnancies with embryo reduction versus expectant management: new data and systematic review. Hum Reprod. (2006) 21:1912–7. doi: 10.1093/humrep/del048, PMID: 16613889

[22] Liu Y Shen Y Zhang H . Clinical outcomes of multifetal pregnancy reduction in trichorionic and dichorionic triplet pregnancies: A retrospective observational study. Taiwanese J Obstetr Gynecol. (2019) 58:133–8. doi: 10.1016/j.tjog.2018.11.025, PMID: 30638467

[23] van de Mheen L Everwijn SMP Haak MC . Outcome of multifetal pregnancy reduction in women with a dichorionic triamniotic triplet pregnancy to a singleton pregnancy: A retrospective nationwide cohort study. Fetal Diagnosis Ther. (2016) 40:94–9. doi: 10.1159/000441650, PMID: 26678498

[24] Stone J Ferrara L Kamrath J . Contemporary outcomes with the latest 1000 cases of multifetal pregnancy reduction (MPR). Am J Obstetr Gynecol. (2008) 199:406.e1–.e4. doi: 10.1016/j.ajog.2008.06.017, PMID: 18928991

[25] Dubé J Dodds L Armson BA . Does chorionicity or zygosity predict adverse perinatal outcomes in twins? Am J Obstetr Gynecol. (2002) 186:579–83. 10.1067/mob.2002.121721 11904627

[26] Yee L Peaceman A Peress D . Evaluation of trichorionic versus dichorionic triplet gestations from 2005 to 2016 in a large, referral maternity center. Am J Perinatol. (2017) 34:599–605. doi: 10.1055/s-0037-1600129, PMID: 28264209

[27] Mansour R Serour G Aboulghar M . The impact of vanishing fetuses on the outcome of ICSI pregnancies. Fertil Steril. (2010) 94:2430–2. doi: 10.1016/j.fertnstert.2010.02.058, PMID: 20400075

[28] Romanski PA Carusi DA Farland LV . Perinatal and peripartum outcomes in vanishing twin pregnancies achieved by *in vitro* fertilization. Obstetr Gynecol. (2018) 131:1011–20. doi: 10.1097/AOG.0000000000002595, PMID: 29742658

