# Correction: Comparing pregnancy outcomes in a population with natural versus surgical reduction of twin pregnancies: a retrospective cohort study

**DOI:** 10.3389/fendo.2026.1856357

**Published:** 2026-04-27

**Authors:** Frontiers Production Office

**Affiliations:** Frontiers Media SA, Lausanne, Switzerland

**Keywords:** spontaneous reduction, twin disappearance syndrome, pregnancy outcome, retrospective cohort study, *in vitro* fertilization

Affiliation “University of Muhammadiyah Yogyakarta, Indonesia” was erroneously given as “University of Münster, Germany” for the Reviewer Supriyatiningsih Wenang.

The original version of this article has been updated.

